# Cross-instrument optical coherence tomography-angiography (OCTA)-based prediction of age-related macular degeneration (AMD) disease activity using artificial intelligence

**DOI:** 10.1038/s41598-024-78327-0

**Published:** 2024-11-07

**Authors:** Anna Heinke, Haochen Zhang, Krzysztof Broniarek, Katarzyna Michalska-Małecka, Wyatt Elsner, Carlo Miguel B. Galang, Daniel N. Deussen, Alexandra Warter, Fritz Kalaw, Ines Nagel, Akshay Agnihotri, Nehal N. Mehta, Julian Elias Klaas, Valerie Schmelter, Igor Kozak, Sally L. Baxter, Dirk-Uwe Bartsch, Lingyun Cheng, Cheolhong An, Truong Nguyen, William R. Freeman

**Affiliations:** 1 Jacobs Retina Center, 9415 Campus Point Drive, La Jolla, CA 92037 USA; 2 Viterbi Family Department of Ophthalmology and Shiley Eye Institute, University of California San Diego, 9415 Campus Point Drive, La Jolla, CA 92037 USA; 3 Division of Ophthalmology Informatics and Data Science, Viterbi Family Department of Ophthalmology and Shiley Eye Institute, University of California San Diego, 9415 Campus Point Drive, La Jolla, CA 92037 USA; 4 Division of Biomedical Informatics, Department of Medicine, University of California San Diego, La Jolla, CA USA; 5 Department of Electrical and Computer Engineering, University of California San Diego, La Jolla, CA USA; 6 Department of Ophthalmology, LMU University Hospital, LMU Munich, Munich, Germany; 7 Department of Ophthalmology, Medical University of Gdańsk, Gdańsk, Poland; 8 The Department of Cognitive Science, University of California San Diego, San Diego, USA; 9 Moorfields Eye Hospital, Dubai, United Arab Emirates; 10 Department of Ophthalmology and Vision Science, University of Arizona, Tucson, USA

**Keywords:** Optical coherence tomography-angiography (OCTA), Cross-instrument training, Artificial intelligence (AI), Deep neural networks (DNN), Age-related macular degeneration (AMD), Predictive markers, Biomedical engineering, Imaging and sensing

## Abstract

This study investigates the efficacy of predicting age-related macular degeneration (AMD) activity through deep neural networks (DNN) using a cross-instrument training dataset composed of Optical coherence tomography-angiography (OCTA) images from two different manufacturers. A retrospective cross-sectional study analyzed 2D vascular en-face OCTA images from Heidelberg Spectralis (1478 samples: 1102 training, 276 validation, 100 testing) and Optovue Solix (1003 samples: 754 training, 189 validation, 60 testing). OCTA scans were labeled based on clinical diagnoses and adjacent B-scan OCT fluid information, categorizing activity into normal, dry AMD, active wet AMD, and wet AMD in remission. Experiments explored cross-instrument disease classification using separate and combined datasets for training the DNN. Testing involved 100 Heidelberg and 60 Optovue samples. Training on Heidelberg data alone yielded 73% accuracy on Heidelberg images and 60% on Optovue images. Training on Optovue data alone resulted in 34% accuracy on Heidelberg and 85% on Optovue images. Combined training data from both instruments achieved 78% accuracy on Heidelberg and 76% on Optovue test sets. Results indicate that cross-instrument classifier training demonstrates high classification prediction accuracy, making cross-instrument training viable for future clinical applications. This implies that vascular morphology in OCTA can predict disease progression.

**Supplementary Information:**

The online version contains supplementary material available at 10.1038/s41598-024-78327-0.

## Introduction

Age-related macular degeneration (AMD) is a major cause of severe visual loss in individuals aged 55 and older^1^. Choroidal neovascularization (CNV), a critical complication of neovascular AMD (nAMD), can cause abrupt and often irreversible vision impairment due to active CNV leakage. Fluorescein angiography has long been the gold standard for detecting CNV leakage^2^. Currently, optical coherence tomography (OCT) and information about intra- or subretinal fluid in the B-scan OCT are crucial for decision-making about the treatment.

Optical coherence tomography angiography (OCTA) is a non-invasive imaging modality that enables visualization of the retinal and choroidal circulation without the need for intravenous dye injection, and it is increasingly being used to detect choroidal neovascularization (CNV) in wet age-related macular degeneration (AMD)^3^. Studies have shown OCTA’s efficacy in identifying choroidal neovascular membranes in nAMD, with sensitivities ranging from 50 to 87% and specificities from 91 to 98% ^4^ compared to the combination of FA with OCT that approaches 100% sensitivity and 97.5–100% specificity for CNV detection^2^.

While OCTA is not currently regarded as the gold standard for decision-making in CNV treatment, its noninvasive characteristics and capability to identify subclinical pathologic changes make it well-suited for diagnostic imaging and monitoring purposes^5^. OCTA can quantify vascular metrics correlating with disease severity and progression and assess morphological changes in CNV vessels in response to anti-VEGF treatment^6^. Recent research^7^ has underscored the constrained ability of en-face Optical Coherence Tomography Angiography (OCTA) images in recognizing choroidal neovascularization (CNV), with OCTA alone encountering challenges in accurately determining CNV activity. To fully harness the potential of OCTA in clinical applications, the development of more effective methods is imperative. Considering the exceptional performance of deep neural networks (DNNs) in surpassing human capabilities in natural image classification^8^, our study concentrates on the creation of deep classifiers for age-related macular degeneration (AMD) stage grading, relying exclusively on en-face OCTA images. These classifiers autonomously extract meaningful features from data, outperforming humans in discerning correlations within various OCTA projections^9^. In our previous work, we hypothesized that vessels seen in OCTA en-face vascular projection could have predictive value for the activity of AMD disease. We have collected, labeled, and classified OCTA scans (Heidelberg Spectralis) into 4 main clinical categories: normal, dry AMD, wet AMD active, and wet AMD in remission. We have shown that AI can achieve 80.36% accuracy on a four-category grading task with solely 2D OCTA en-face vascular projections from Heidelberg Spectralis and the overall AI classification prediction of disease activity was significantly better than the 60% of masked human experts prediction^9^. However, it is important to mention that this value was tested on a smaller and segmentation error-free test set. In this paper, we work on a larger test set that may have some minor segmentation errors (error-prone test-set). As a continuation of this research, we wanted to expand the existing dataset and add a new OCTA dataset from a different camera to enable cross-instrument training for OCTA-based AMD disease activity prediction.

Domain shift is a significant hurdle for deep learning, occurring when models trained on a specific dataset perform poorly on varied datasets from different hospitals, imaging protocols, and manufacturers^10^. This challenge is exemplified by the first commercial AI-powered device for diabetic retinopathy screening, which was FDA-approved only for one specific camera type, highlighting concerns about performance degradation due to domain shift^11^.

The purpose of this work is to evaluate the effectiveness of predicting AMD disease activity using a cross-instrument training dataset from two different OCTA cameras, Heidelberg and Optovue. By expanding our Heidelberg Spectralis dataset and adding data from Optovue Solix, we aim to improve training diversity and accuracy. Our goal is to develop an algorithm that can predict disease activity regardless of the imaging instrument used.

## Methods

### OCTA data selection and labeling

This study, a cross-sectional retrospective analysis conducted at the Jacobs Retina Center, Shiley Eye Institute, University of California San Diego, California, USA, was granted approval by the Institutional Review Board (IRB#120516) for the review of the patient’s chart and images. Hence, the informed consent was waived for retrospective chart review. The study was conducted in accordance with the ethical principles outlined in the Health Insurance Portability and Accountability Act of 1996. Adhering to the tenets of the Declaration of Helsinki, all data collection procedures prioritized patient anonymity and confidentiality.

A total of 1478 high-resolution (OCTA) en-face 3 × 3 mm scans acquired with Heidelberg Spectralis were retrospectively collected from patients treated at the Jacobs Retina Center between 2021 and 2023, from the Department of Ophthalmology, University Hospital, Ludwig-Maximilians-University, and from Moorfields Eye Hospital UAE. Clinical data, including imaging reports and electronic health records managed through Epic (Epic Systems, Verona WI, USA) and EMR: Pragmedic Solutions (Oakbrook Ter, IL, U.S.A), were meticulously reviewed to extract relevant clinical information such as diagnosis and details of anti-vascular endothelial growth factor (anti-VEGF) therapy. In addition to the Heidelberg OCTA dataset, we collected the Optovue Solix OCTA (Visionix) dataset from Medical University Gdańsk, Poland that comprised 1003 high-resolution 6 × 6 mm OCTA scans.

Inclusion criteria for both Heidelberg and Optovue datasets encompassed patients aged over 50 years with a confirmed diagnosis of age-related macular degeneration (AMD) and the availability of high-quality OCTA scans devoid of motion artifacts or background noise, with a quality index (Q) exceeding or equal to 25 ^12^ in images acquired with Heidelberg Spectralis and Scan Quality exceeding or equal to 7 (in the quality scale from 0 to 10) in the scans obtained with Optovue Solix OCTA. Both datasets included healthy, age-matched subjects as normal controls. Scans with CNV of an etiology different from AMD as well as scans with motion artifacts, incomplete scans, scans with background noise, and poor quality due to media opacity (with quality index Q < 25 in Heidelberg Spectralis and < 7 in Optovue Solix) were excluded. Each eye was analyzed independently and OCTA scans as well as exclusion and inclusion criteria were performed per eye level. Patients were categorized into four groups according to their clinical classification. Group 1, labeled as “wet active AMD,” comprised patients with (CNV) and fluid observed in B-scan OCT scans, including those newly diagnosed and treatment-naïve individuals, as well as patients undergoing anti-VEGF treatment with fluid detected on OCT. Group 2, designated as “wet AMD in remission,” consisted of patients with treated CNV but without fluid evident on OCT scans. Group 3 encompassed cases of dry AMD without CNV or fluid in OCT scans, while Group 4 comprised healthy eyes devoid of AMD or any other retinal pathology^9^.

Clinical activity was determined by two retina specialists based on the presence of subretinal and/or intraretinal fluid observed in OCT B-scans obtained on the same day as the OCTA scan. This determination relied on information obtained from electronic chart reviews, including diagnosis and anti-VEGF treatment, without direct observation of the OCTA images, establishing it as the “gold standard” for labeling. Any discrepancies in labeling were resolved through review by a senior retina specialist (W.R.F.), who made the final diagnosis after assessing the imaging results.

SD-OCT and OCTA centered at the macula using the Heidelberg Spectralis HRA + OCT (Heidelberg Engineering, Heidelberg, Germany) examinations were conducted, and the OCTA scan parameters were standardized as follows: 512 B-scans, pattern size of 3 × 3 mm/10° × 10°, with a 6 mm interval between B-scans, and an average of 5 ART images. Segmentation correction was performed using the automated segmentation provided by the embedded Heidelberg software. The OCTA scans were exported solely as 2D en-face vascular projections (superficial, deep, avascular, and choriocapillaris layers in case of Heidelberg Spectralis). Prior to generating the vessel OCTA image, the automated segmentation from the Heidelberg software underwent review by two retina specialists, who were also responsible for labeling the data. Each scan was meticulously examined for segmentation errors directly on the Heidelberg SPECTRALIS machine using Heyex-2 software, by scanning through the 512-line B-scans. Specifically, the integrity of the Bruch membrane line was assessed, while the inner retinal segmentation was not reviewed.

**Fig. 1:**
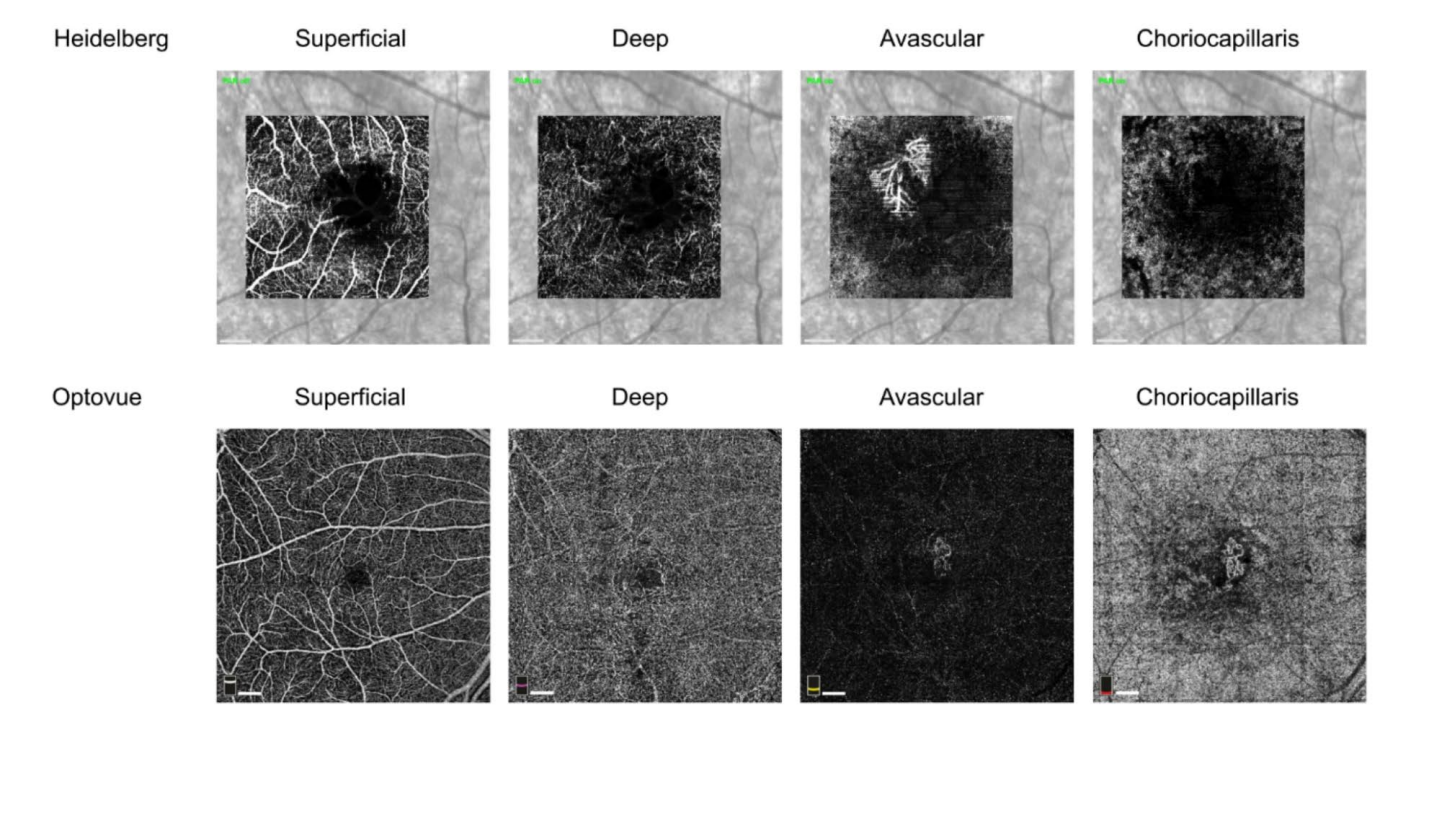
Examples of OCTA images in the “active” category of AMD from different patients for Heidelberg and Optovue instruments. Both images show neovascularization (CNV) in the avascular slab for Heidelberg (3 × 3 mm image size) and outer retina and choriocapillaris slab on Optovue (6 × 6 mm image size) example. Note the difference in the field of view of vascular projections in both cameras and the presence of IR background image in Heidelberg Spectralis images. The white bar on the left lower corner of each image represents a scale bar of 500 μm.

If any segmentation errors, whether major or minor, were detected in the Bruch membrane, corrections were made accordingly and annotated as “Y” for major errors or “M” for minor errors within an Excel spreadsheet. Instances where segmentation was accurate and exhibited proper delineation of the retinal layers were denoted as “N” for no error. Similar methods were applied to the data collected at the Medical University of Gdansk, Poland with the Optovue Solix (Visionix, USA) camera in terms of segmentation and data labeling. The field of view of scans obtained with Optovue Solix was larger than with Heidelberg. Patients obtained OCT and OCTA scans with Optovue Solix with the following parameters: 512-line B-scans, scan size 6 × 6 mm/ 20° × 20°. Each exported OCTA scan is composed of 4 en-face projections: Angio-Superficial, Angio-Deep, Angio-Outer Retina and Angio-Choriocapillaris. Figure 1 shows vascular en-face 2D projections of “active, wet AMD” category of vessels from different patients imaged with Heidelberg Spectralis and Optovue Solix instruments.

### Artificial intelligence methods

We split the data into training/validation/testing subsets: for Heidelberg Spectralis data (1478 samples: 1102 for training, 276 for validation, 100 for testing) and for Optovue Solix data (1003 samples: 754 training, 189 validation, 60 for testing). The testing samples from Heidelberg Spectralis and from Optovue Solix were unseen data for AI. Our data split was performed at the eye level, not at the patient level. Each eye was analyzed independently, and the exclusion criteria were applied at the eye level. This is consistent with clinical practice, as it is possible for each eye to have a different diagnosis, and the condition of one eye does not allow for accurate prediction of the clinical state of the fellow eye with high certainty. Furthermore, we ensured that no scans from the same eye at different time points were split between the training and testing sets, which avoids any potential data leakage.

**Figure 2:**
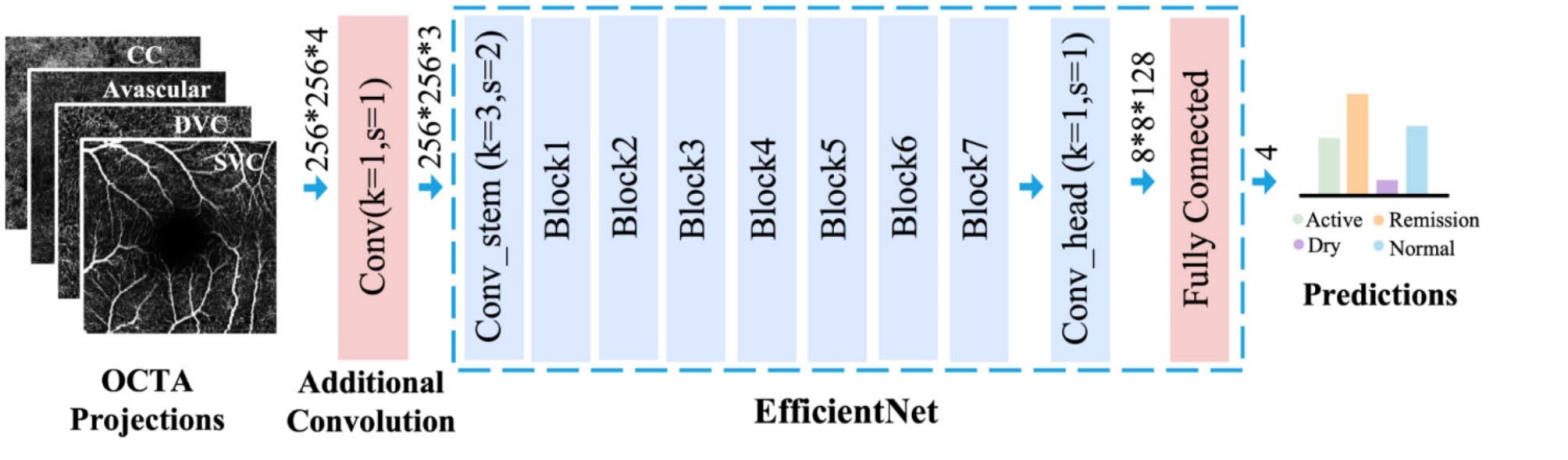
The network structure of the classifier used in our experiments. We use an ImageNet pre-trained EfficientNet-b5 as the backbone with a few modifications. An additional convolution layer is inserted to handle the four-channel inputs of OCTA and the output channel of the final fully connected layer’s output channels are reduced to four, aligning with the number of our desired categories.

Since the focus of this study is the interaction of OCTA projections from different instruments, we utilize the deep classifier proposed in our previous work^9^ with a few modifications. In detail, we initially trained the neural network using a popular natural image dataset, ImageNet^13^, and then refined its performance to suit our specific OCTA projection images, Heidelberg only, Optovue only, or a mixture of both. We utilized EfficientNet-b5 ^14^ as the backbone with necessary modifications to accommodate the unique characteristics of OCTA data. Specifically, given that EfficientNet typically handles three-channel inputs corresponding to RGB in natural images, while our OCTA data comprises four projections at different depths, we incorporated an additional convolution layer with a kernel size of one to manage the four-channel inputs (Fig. 2). Additionally, we reduced the output channel of the final fully connected layer to four, aligning with the relevant categories for our task (active, remission, dry, and normal). Notably, unlike our previous method^9^, we opted not to reduce the output channel of the last convolution layer or implement a warm up strategy, as these tactics did not enhance the final performance given the current data amount.

In preparing the training data, we downsampled each OCTA projection from 512 × 512 to 256 × 256 and applied various augmentation techniques to enhance diversity. These techniques included random flipping, rotation, cropping with resizing, gamma transformation, Gaussian smoothing for the diversity of intensity, and grid distortion to augment the shapes. We also adopted a sample-wise normalization to whiten the sample intensity. We implemented our deep classifiers on the PyTorch platform. During training, we utilized the Adam optimizer with 10 − 5 weight decay. The initial learning rate was set to 10 − 3 and decreased by a factor of 10 after 400, 800, and 1200 epochs of training, during a total of 1500 epochs training. Instead of the cross entropy loss, a cosine loss function^15^, which has been proven effective for small datasets, was used as our optimization target. Also, oversampling training data in each category was used to balance their distribution. For validation, we conducted a 5-fold experiment, resulting in five classifiers, whose ensemble predictions were considered the final results. Please refer to the “Supplementary Figure S1” for the images of confusion matrices and detailed descriptions.

## Results

### Artificial intelligence classifier performance

Table 1 shows the data characteristics with numbers for OCTA samples from both cameras, with numbers of patients, eyes, and numbers of visits.

**Table 1:** Data characteristics for OCTA images from Heidelberg and Optovue including total number of patients, number of patients with one eye imaged, number of patients with both eyes imaged, total number of scans/eyes, and number of visits. N- number.

Camera OCTA	Total *N* of patients /unique names	*N* of patients with one eye imaged	*N* of patients with both eyes imaged	Total *N* scans /eyes	Eyes with 1 visit	Eyes with 2 visits	Eyes with 3 visits	Eyes with 4 visits	Eyes with 5 visits	Eyes with 6 visits and more
Heidelberg	525	226	299	1478	602	103	37	30	12	49
Optovue	736	500	236	1003	661	93	33	5	5	2

In Table 2, we present the results of overall prediction accuracy for two different datasets separately and combined. Training the OCTA data solely on Heidelberg yielded a 73% overall prediction accuracy for the disease category on the Heidelberg test set, and 60% accuracy on the Optovue test set. Training on Optovue data only resulted in 34% overall accuracy on Heidelberg, and 85% accuracy on Optovue test sets. Combining training data from both instruments (Heidelberg + Optovue) achieved 78% overall accuracy on Heidelberg and 76% on Optovue test sets.

**Table 2:** The overall prediction accuracy for both different OCTA instruments and datasets used separately and combined.

Data source (training set)	Performance metrics on Heidelberg (100 samples test set)	Performance metrics on Optovue Solix (60 samples test set)
	Accuracy	Accuracy
Heidelberg Spectralis	0.73	0.60
Optovue Solix	0.34	0.85
Heidelberg + Optovue	0.78	0.76

**Fig. 3:**
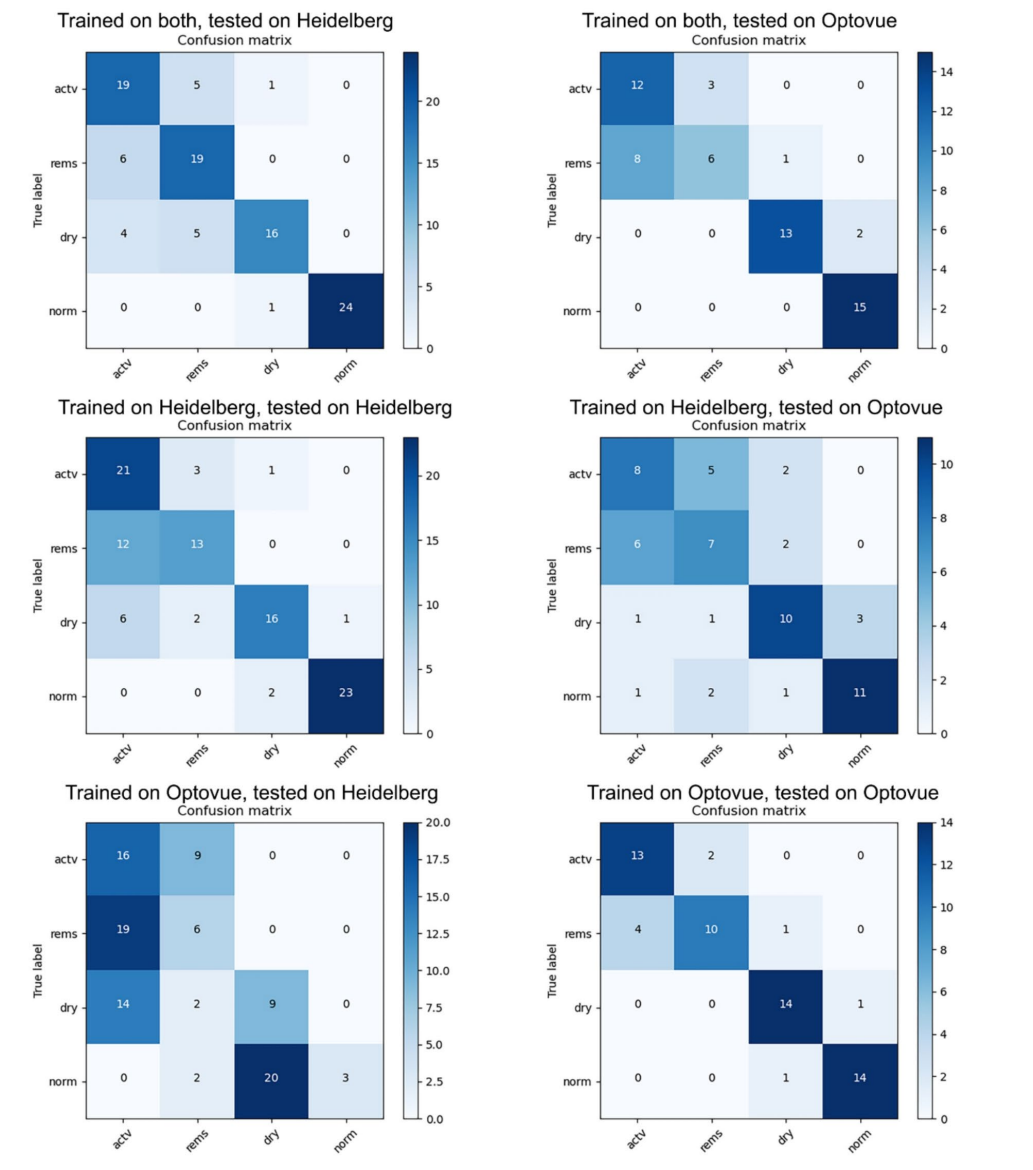
Confusion matrices for each combination of training and testing set for each clinical category (active, remission, dry AMD, and normal).

Utilizing these confusion matrices (Fig. 3), we calculated the sensitivity and specificity values for the four clinical categories for Heidelberg test set (100 samples) and Optovue test set (60 samples) depending on the training set (data source: either Heidelberg Spectralis, or Optovue Solix or both Heidelberg and Optovue Solix combined). Table 3 shows performance metrics (sensitivity and specificity) for Heidelberg test set and Table 4 presents performance metrics (sensitivity and specificity) for Optovue test set.

**Table 3:** Performance metrics on Heidelberg test set (100 samples) depending on clinical classification and training set (data source).

	Performance metrics on Heidelberg (100 samples test set) depending on clinical classification and training set							
	Active wet AMD		Remission AMD		Dry AMD		Normal	
Data source (training set)	SN	SP	SN	SP	SN	SP	SN	SP
Heidelberg (H)	0.84	0.76	0.52	0.93	0.64	0.96	0.92	0.99
Optovue (O)	0.64	0.56	0.24	0.83	0.36	0.73	0.12	1.00
H + O	0.76	0.87	0.76	0.87	0.64	0.97	0.96	1.00

H-Heidelberg, O-Optovue, SN- Sensitivity, SP-Specificity.

**Table 4:** Performance metrics on Optovue test set (60 samples) depending on clinical classification and training set (data source).

	Performance metrics on Optovue (60 samples test set) depending on clinical classification and training set							
	Active wet AMD		Remission AMD		Dry AMD		Normal	
**Data source (training set)**	SN	SP	SN	SP	SN	SP	SN	SP
Heidelberg (H)	0.53	0.82	0.47	0.82	0.67	0.89	0.73	0.93
Optovue (O)	0.87	0.91	0.67	0.96	0.93	0.96	0.93	0.98
H + O	0.80	0.82	0.40	0.93	0.87	0.98	1.00	0.96

H-Heidelberg, O-Optovue, SN- Sensitivity, SP-Specificity.

To investigate the impact of field of view of OCTA on the accuracy of prediction we generated synthetic 3 mm × 3 mm Optovue images by centrally cropping the original 6 mm × 6 mm Optovue images for both the training and testing sets. Following the previously established training strategy, we trained a new classifier on the 3 mm × 3 mm data. We then evaluated the performance of this classifier, along with the original 6 mm × 6 mm classifier, on both the 3 mm × 3 mm, and 6 mm × 6 mm test sets. The results are presented in Table 5.

**Table 5:** Comparison of classifier performance on 3 mm × 3 mm and 6 mm×6 mm optovue images.

	Test on 6 × 6 mm	Test on 3 × 3 mm
Train on 6 × 6 mm	0.85	0.6333
Train on 3 × 3 mm	0.6833	0.7333

## Discussion

In our study, we aimed to train the algorithm to predict the disease activity of AMD using a cross-instrument training dataset derived from two different manufacturers and OCTA cameras, such as Heidelberg and Optovue Solix (OCTA) images. We propose to create an algorithm that will be able to predict the disease activity independently from the instrument used for imaging.

We have shown that our neural network can predict disease activity with high accuracy for both OCTA instruments, but the performance between these two cameras varies. We achieved 73% accuracy for Heidelberg data when the images from the Heidelberg camera were used for training. This performance was improved to 78% when images from both cameras (Heidelberg and Optovue) were used for training. On the other hand, the overall prediction accuracy of the algorithm trained on data sourced from the Optovue Solix camera was 85%, which was higher than the 73% of Heidelberg’s performance when trained on the same source. Notably, when we combined both data sources from Heidelberg and Optovue cameras for training and tested it on the Optovue test set, the performance did not increase like in the case of Heidelberg data, but it dropped substantially from 85 to 76%. The reason for this result can be attributed to two main factors. Firstly, our previous work has shown that the Heidelberg data contains category-dependent segmentation errors, which significantly affected classifier training. Introducing Heidelberg data into the training set of Optovue data complicates the problem, resulting in suboptimal performance. Secondly, these two OCTA datasets have different fields of view (FoV). Heidelberg Spectralis uses 3 × 3 mm images, while Optovue scans have a larger FoV of 6 × 6 mm. Consequently, Optovue data includes areas that are not present in Heidelberg data. Augmenting Heidelberg with Optovue data enhances prediction accuracy, as the additional Optovue data enlarges the training set. However, augmenting Optovue with Heidelberg data has the opposite effect, as the missing areas in Heidelberg data force the classifier to ignore useful cues present only in Optovue images, complicating the task.

The limitation of the study is the discrepancy in FoV between the cameras which is due to the different imaging protocols used by the main centers providing the data. The preferred pattern for Heidelberg Spectralis was 3 × 3 mm, while for the external Optovue Solix dataset, it was 6 × 6 mm. Future studies should compare performance using identical imaging protocols with matching FoVs for a fair comparison. However, this discrepancy reflects real-world conditions, where diverse manufacturers and imaging protocols are common, highlighting the practical relevance of our work.

Another important aspect influencing performance is the presence of data from the same camera in both the training and test sets. It is intuitive and expected that classifier performance will be poorer if trained on data sourced from a different camera than the test set. In our study, when Optovue images were used exclusively for training, the performance on Heidelberg data showed only 34% accuracy, significantly lower than the masked human experts’ prediction of 60% from our previous work. Conversely, when tested on the Optovue set, despite being trained solely on Heidelberg data, the performance remained at 60%, which is twice as good as for Heidelberg. This outcome is expected due to two reasons: (1) The Heidelberg dataset has more training samples (1478 vs. 1003). With 47.4% more training data, the classifier trained on the Heidelberg set is less likely to overfit and generalizes better; (2) As discussed previously, the larger FoV of Optovue data provides more contextual information. In Table 5 of results we observe, that the accuracy improves when the field of view (FoV) of the test set matches that of the training set, which is a common characteristic of all data-driven models. Second, we observed that classifiers trained with a larger FoV performed better, indicating that peripheral information is beneficial. This is further supported by the comparison 0.6333 < 0.6833: training with peripheral data and testing on samples without the periphery results in a greater performance drop. In summary, peripheral data provides useful information for AMD grading.

In our analysis, we examined prediction accuracy for specific clinical categories. The highest sensitivity and specificity for both datasets were achieved for dry and normal categories, while the lowest sensitivity was observed in remission and active categories. This is consistent with our previous findings^9^. The classifier more easily distinguishes dry and normal categories due to the absence of pathological vessels in the avascular and choriocapillaris layers. However, distinguishing active wet AMD vessels from those in remission is challenging due to varying patterns of pathological vessels in these layers. For human observers, features of active vessels in CNV were previously described by Coscas et al.^16^. Future studies should explore explainable AI to understand how the classifiers can better distinguish these vessels as human observers.

The lower success rate in predicting the “active” and “remission” categories could be attributed to several limitations observed in OCTA as an imaging modality, including the unchanged nature of CNV vasculature in some patients despite the absence of leakage. Slow flow or vessel pooling in some cases may evade detection. Sensitivity and specificity for CNV detection using OCTA have been reported variably across different studies.^17,18^. Despite expectations based on other imaging modalities such as FA and SD-OCT, CNV vessels may not always be observed in OCTA, particularly in cases classified under the remission category of nAMD, even in patients treated with anti-VEGF therapy who show no fluid in SD-OCT. Various hypotheses have been proposed to explain the persistence of CNV vessels in OCTA despite anti-VEGF treatment, suggesting differences in the structure and responsiveness to therapy among different components of CNV vasculature^18^. Direct visualization of CNV using OCTA offers insights into the pathophysiology of neovascular AMD, potentially impacting treatment decisions.

Our algorithm demonstrates relatively high sensitivity and specificity in predicting the clinical status of AMD patients based solely on OCTA vascular en face projections in both used instruments (Heidelberg and Optovue) making the perspective of cross-instrument training possible for future clinical applications. This implies that vascular morphology could serve as a predictive indicator of disease progression and warrants investigation across various stages of neovascular AMD and under different treatment regimens using different OCTA imaging cameras.

Although both Heidelberg Spectralis and Optovue are widely used for OCTA imaging, the limitation of our study is that the algorithm was tested only on these two instruments. In the future, we want to expand our dataset and provide a new dataset acquired with different OCTA instruments, such as Topcon Triton, Topcon Maestro2, or Zeiss Plex Elite 9000 to broaden data training diversity and enhance accuracy for AMD disease activity prediction as well as to train the models for different diagnosis such as i.e. diabetic retinopathy. The main challenge is to collect a large amount of standardized, labeled imaging data, as there is a scarcity of open databases with high-quality OCTA scans labeled scans. This problem requires additional labeled data from the new setting, which is challenging to obtain, and time-consuming along with additional resources to fine-tune the model on new labeled data. The value of our internal dataset is that it was meticulously reviewed, labeled, and in the presence of segmentation errors of Bruch’s membrane, manually corrected by experienced retina specialists. It is challenging to obtain similar datasets and the collection process is very time-consuming. This study introduces a novel approach to cross-instrument neural network training for OCTA, utilizing images from two distinct cameras with differing imaging protocols and fields of view to predict AMD disease activity solely from 2-D OCTA vascular projections. Our previous work^9^ showed high accuracy in the prediction of the AMD disease, but it comprised a total of 310 images acquired with one camera Heidelberg Spectralis, while in the current study, we expanded the Heidelberg dataset to 1478 images and added the new dataset of Optovue Solix of a total 1003 OCTA samples for a cross-instrument training experiment. We have shown overall high classification prediction accuracy for both Heidelberg and Optovue data with some performance variation depending on the source (imaging device) of training data. To date, there are no studies solely using OCTA for AMD diagnosis. Instead, current work typically relies on color fundus imaging, fluorescein angiography (FA), and more recently, OCT imaging. Alqudah et al. trained a custom CNN to classify AMD into five distinct stages using OCT B-scans^19^. Other studies have utilized multimodal imaging, including fundus photographs, OCT B-scans, and OCTA projections, for AMD detection and grading.^20–22^.

## Conclusions

Our model exhibits potential for automating disease diagnosing and aiding decision-making processes in nAMD, serving as a valuable tool independently from the manufacturer or camera used for imaging. Further studies are needed to improve the performance of the algorithm on the data from different sources and expand it for other OCT and OCTA manufacturers on the market to enable the cross-instrument prediction of AMD disease to a fuller extent and independently from imaging protocol.

## Electronic supplementary material

Below is the link to the electronic supplementary material.

Supplementary Material 1

## Data Availability

The datasets generated and/or analyzed during the current study are not publicly available due to the possibility of patients’ identification from retinal imaging of OCTA vascular patterns but are available from the corresponding author upon reasonable request. For some institutions, this might require a Data Sharing Agreement.
