# Supplementary material for: Cross-instrument optical coherence tomography-angiography (OCTA)-based prediction of age-related macular degeneration (AMD) disease activity using artificial intelligence
Source: Sci Rep. 2024 Nov 7;14:27085. doi: 10.1038/s41598-024-78327-0 (PMC11544254; doi:10.1038/s41598-024-78327-0)

Trained on both, tested on Heidelberg

Validation 1 | Validation 2 | Validation 3 | Validation 4 | Validation 5 | Mean | Variance

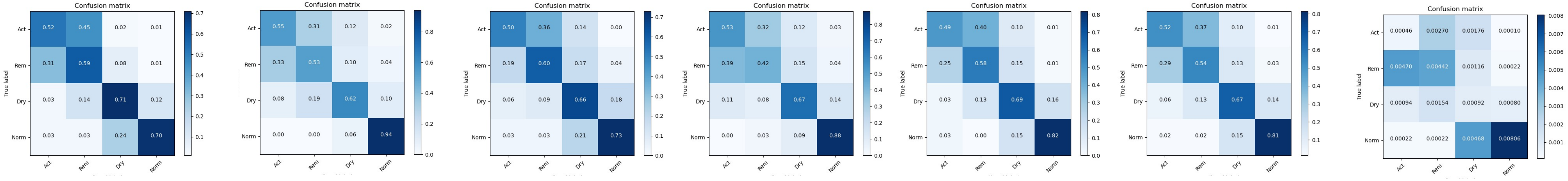

Trained on both, tested on Optovue

Validation 1 | Validation 2 | Validation 3 | Validation 4 | Validation 5 | Mean | Variance

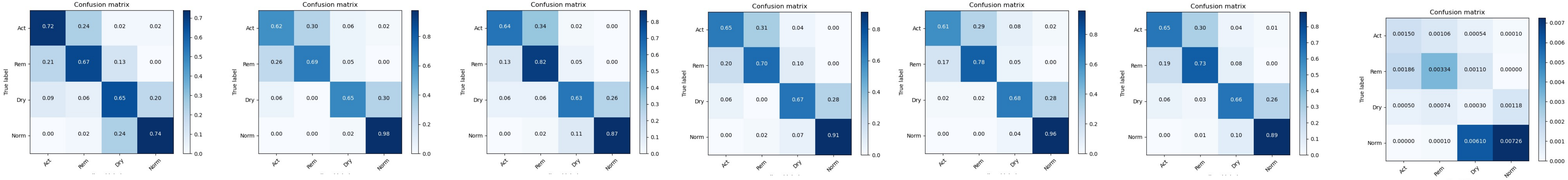

Trained on Heidelberg, tested on Heidelberg

Validation 1 | Validation 2 | Validation 3 | Validation 4 | Validation 5 | Mean | Variance

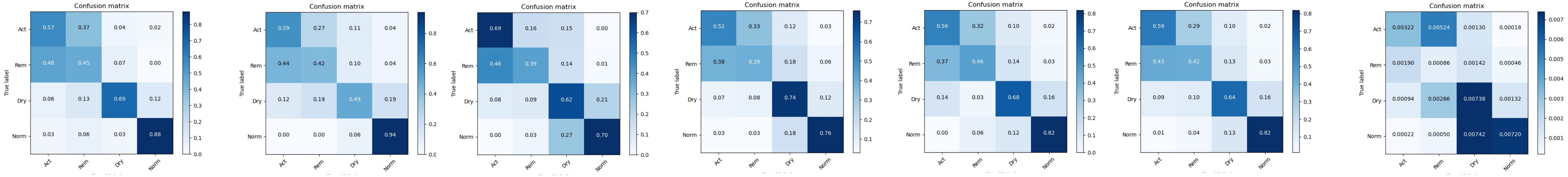

Trained on Heidelberg, tested on Optovue

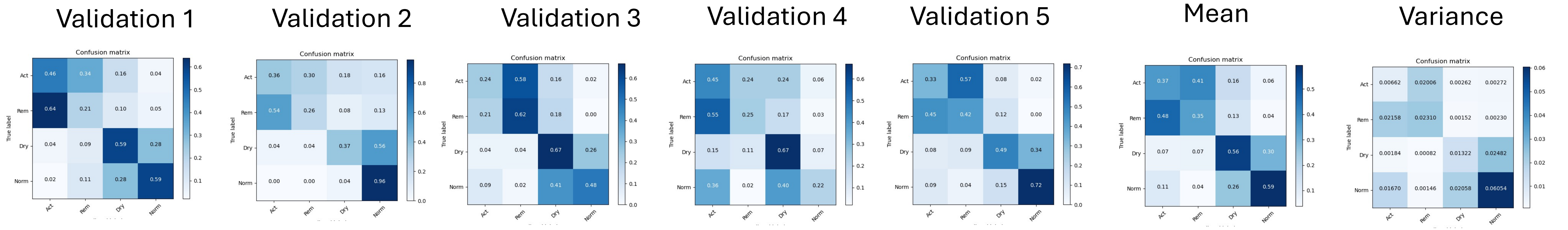

Trained on Optovue, tested on Heidelberg

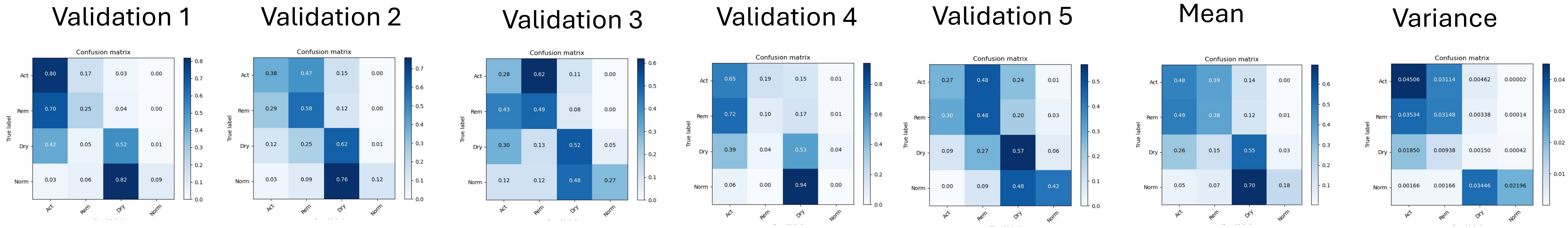

Trained on Optovue, tested on Optovue

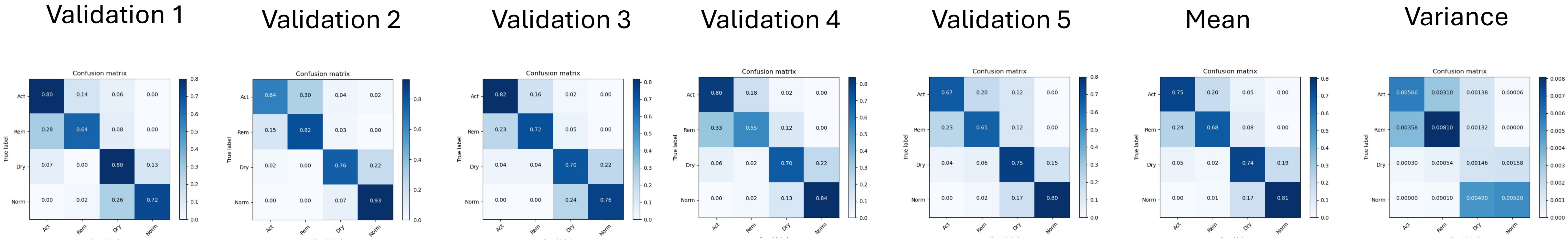

Supplement: Supplementary file 1 — Supplementary Material 1 [file 41598_2024_78327_MOESM1_ESM.pdf]
